# When and How Does Psychological Voice Climate Influence Individual Change Readiness? The Mediating Role of Normative Commitment and the Moderating Role of Work Engagement

**DOI:** 10.3389/fpsyg.2017.01737

**Published:** 2017-10-09

**Authors:** Chun-Hsien Lee, Mei-Ling Wang, Min-Shi Liu

**Affiliations:** ^1^Graduate Institute of Human Resource and Knowledge Management, National Kaohsiung Normal University, Kaohsiung, Taiwan; ^2^Department of Business Administration, Tamkang University, New Taipei City, Taiwan; ^3^Department of Business Administration, Soochow University, Taipei, Taiwan

**Keywords:** psychological voice climate, normative commitment, individual change readiness, work engagement, social identity theory, public sector

## Abstract

This research explores the linking mechanisms and conditional processes underlying the relationship between psychological voice climate and individual change readiness. In accordance with the social identity theory, we argued that normative commitment would mediate the relationship between psychological voice climate and individual change readiness; furthermore, work engagement would moderate the proposed indirect effect. Two-wave survey data were collected from 187 full-time employees in a government-owned institute of research and development and were adopted for moderated mediation analysis. The results showed that normative commitment mediates the relationship between psychological voice climate and individual change readiness. Furthermore, work engagement strengthens the effect of psychological voice climate on individual change readiness in an indirect manner via normative commitment. Based on the findings, the theoretical implications and practical suggestions were discussed.

## Introduction

Faced with financial austerity and economic crisis, many public sectors of Western countries have turned to reforms aimed at cutting back on expenses and improving efficiency (van der Voet and Vermeeren, 2017). The literature on cutback management suggests that cutbacks may result in decreased job satisfaction and morale as well as increased work-related stress and intention to leave (Raudla et al., 2015). Due to the high failure rate of organizational change, researchers have made efforts to explore critical factors that may contribute to the successful implementation of organizational change (Rafferty et al., 2013). Starting from the notion that successful organizational change mainly depends on generating employee support and enthusiasm for proposed changes, rather than merely overcoming change resistance (Piderit, 2000), we concentrate our study around the concept of attitudes toward change. We argue that, although public bureaucracies are often viewed as unresponsive to reforms or strongly resistant to change, public organization leaders or change agents can considerably increase the success rate of their change initiatives by gleaning insights into key antecedents of employees’ attitudes toward organizational change.

Change readiness is one of the most prevalent positive attitude toward change that has been addressed in the literature of organizational change. By definition, individual change readiness reflects the extent to which an individual is inclined to accept, embrace, and adopt a particular way to change the current situation purposefully (Holt et al., 2007). Regarding the necessity and inevitability of change, organizations are thus encouraged to consider employee readiness factors in the implementation of change initiatives (Eby et al., 2000; Cunningham et al., 2002). Along with the essence and relevance of individual readiness in the organizational change context, most research has focused on the manners in which change initiatives have been launched and implemented (Oreg and Sverdlik, 2011) or has examined antecedents such as managerial support for the change and employee change efficacy in change competence (Eby et al., 2000; Rafferty and Simons, 2006). Such a perspective assumes that, during organizational change, certain buttons must be pressed to induce positive employee responses to change. However, instead of focusing on change-specific drivers of employee attitudes toward change, careful consideration of the internal context in which the organizational change occurs is required to ensure the success of change implementation (Herold et al., 2007). In other words, the pre-change internal context becomes important in fostering constructive employee responses to organizational change (van der Voet and Vermeeren, 2017).

Tierney (1999) viewed the psychological climate, including dimensions of trust, participation, and support, as preconditions for an environment conducive to change. Similar to the concept of psychological climate, psychological voice climate involves employee perceptions to participate in organizational decisions by having the opportunity to advance their ideas and have them considered honestly by their employer (Farndale et al., 2011). Applying Blau’s (1964) exchange theory, a positive voice climate generates long-term positive attitudes toward the organization because employees feel recognized, heard, and trusted by their immediate manager as well as the organization. Devos et al. (2007) found that, when employment relationships are characterized by high levels of mutual trust, employees are more open to organizational change. In line with van den Heuvel et al.’s (2017) argument on the internal context, we focus on the concept of voice climate to describe the internal context in which an organizational change takes place.

Prior research indicates that, when employees are frequently and consistently asked their opinions and offered opportunities to provide suggestions about work-related issues, they are likely to have greater commitment toward their organization (Farndale et al., 2011). Empirical studies have revealed that employees report greater change readiness when they feel emotionally attached to the organization (McKay et al., 2013). Recent studies have also increasingly paid attention to the way how the particular context of public organizations may influence the implementation of organizational change (Kickert, 2014; van der Voet et al., 2015). For instance, rather than affective commitment, normative commitment (i.e., the sense of obligation to remain with the organization) was found to be more important in public sectors than private sectors due to the nature and content of the written employment contracts and implicit psychological contracts (Boyne, 2002; Markovits et al., 2007). Moreover, normative commitment is relevant to the levels of individual change readiness that involve a sense of moral duty to do the right thing, as expected by the organization. This duty proceeding from the interiorized norm of reciprocity is important for change-relevant attitudes; as such, normative commitment should be integrated into a model of individual change readiness targeting public sector employees. Regarding the characteristics of employment in the pubic organizations, it is essential to develop the model specific for public sector employees. Thus, the first purpose of the study is to answer the important question—namely, whether the existence of psychological voice climate can enhance public sector employees’ change readiness underlying the mechanism of normative commitment.

On the other hand, organizational change may become excessive when its demands exceed the employees’ resources to cope with the impact of organizational change, thereby provoking negative reactions to change (Johnson, 2016). Under the circumstances, engaged employees are expected to continuously put a lot of energy into their work and keep concentrated on what they are doing until the job is complete (Gordon et al., 2015). Work engagement has also been suggested to enhance positive organizational change (Avey et al., 2008). In addition, work engagement has been found to increase employee effectiveness in achieving the organizational goals (Mackay et al., 2017). However, very few studies—with a limited number of exceptions—have examined the moderating role of work engagement in translating a pre-change work context (i.e., psychological voice climate) into a supportive response toward organizational change (i.e., individual change readiness) in public organizations. Thus, the second purpose of this study is to explore whether work engagement enhances the indirect effects of psychological voice climate on individual change readiness.

**FIGURE 1:**
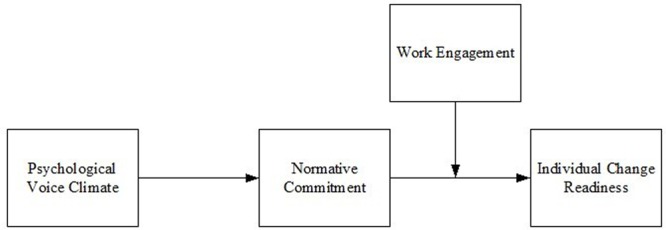
Hypothesized model.

The current research is expected to contribute to the literature on voice climate, normative commitment, and individual change readiness in several ways. First, the research is the first to clarify how psychological voice climate influences individual change readiness through normative commitment. In this way, our knowledge demonstrates the importance of pre-change internal contexts for public organizations. Second, the research utilizes the social identity theory to clarify how psychological voice climate affects individual change readiness by revealing the mediation of normative commitment. Third, by investigating the moderating role of work engagement on the indirect effect of psychological voice climate on individual change readiness through normative commitment, the research makes contributions to the engagement literature by identifying the effectiveness of work engagement interventions. The proposed theoretical model is illustrated in **Figure 1**.

## Theories and Hypotheses

### Psychological Voice Climate and Normative Commitment

The psychological voice climate determines the extent to which employees perceive that they are encouraged to display voice behavior in public. Existing literature (Morrison and Milliken, 2000; Morrison et al., 2011) defines voice climate as referring to employees’ beliefs about whether a particular context is safe for them to speak up on suggestions as well as how effective their voice will be heard and acted upon.

Of the three components of organizational commitment, a less common—but equally feasible—view of commitment is normative commitment, which refers to the employee’s belief that it is the individual’s responsibility or obligation to be loyal to the organization (Meyer and Allen, 1991). Employees with greater normative commitment feel that they ought to keep staying in the organization (Meyer and Allen, 1997). According to social exchange theory, the exchange benefit includes not only the tangible goods and services, but also the intangible prestige, approval, status, and recognition, which are socially valued (Blau, 1964; Tekleab and Chiaburu, 2011).

Normative commitment rises as a result of a moral duty to repay the organization for benefits received from the organization itself or the socialization experiences that emphasize the importance and necessity of keeping loyal to the employer (Yucel et al., 2014). In addition to the observation of role models or the contingent use of rewards and punishment, a more specific reciprocity mechanism may operate in the development of normative commitment (Meyer and Allen, 1991). For example, the receipt of special favors from the organization may constrain employees to stay even when the organization is experiencing external or internal pressure for reforms or change. Yucel et al.’s (2014) study of top management teams indicated that CEO leadership both directly and indirectly enhanced employees’ and top executives’ normative commitment.

The current study seeks to extend existing research in this field by investigating the effect of positive voice climate on public sector employees’ normative commitment. Employees usually consider normative commitment as a moral imperative based on social norms or prior socialization experiences occurring in the organization (Meyer et al., 2006). Public sector organizations operate and function as traditional bureaucracies and tend to put emphasis on the importance of standardized procedures and formality. When public sector employees enter into the work environments, they are not necessarily expected to provide proactive suggestions or participate in work-related decisions. Under the circumstances, if public sector employees are permitted to make suggestions concerning work-related issues without experiencing any negative consequences to their status or career, and their ideas are listened to and adopted to improve organizational effectiveness, they are likely to feel recognized and valued by their organization. Once public sector employees experience such participation, involvement, and recognition of the internal environment, their stereotypical perception or image of a public sector organization breaks down. In turn, this psychological motivation leads to a feeling of obligation to return the favor by staying with the organization.

Additionally, social identity theorists argue that in some social situations, individuals think of themselves and others in terms of a particular group membership (Tajfel, 1978). When individual is surrounded by the atmosphere of expressing their own opinions and suggestions to improve the environment and feel recognized by other members, they are likely to identify with the group or the organization, and then produce the obligation to stay with the organization. More specifically, public sector employees’ psychological voice climate makes them more positively feel committed to and involved with the organization. When surrounded by a positive voice climate, public sector employees will pay back these benefits to the organization through enhanced normative commitment. Based on the above discussion, we propose that:

Hypothesis 1: Psychological voice climate will be positively related to normative commitment.

### Mediation of Normative Commitment

Individual change readiness is often viewed as one kind of attitudes affected by the content and process (Armenakis and Bedeian, 1999; Holt et al., 2007; Walker et al., 2007). An individual, who is ready to change, will exhibit a proactive and positive attitude toward the change, which can be translated into a willingness to support the change and a feeling of confidence in the success of the change. Thus, the root of individual change readiness lies with those employees who accept, embrace, and are willing to carry out a specific plan to change the status quo and do so intentionally (Holt et al., 2007).

It has been widely argued that an organization gives priority to motivating employee change readiness when implementing any change initiatives (Self et al., 2007). Several studies concentrating on the motivations of public sector employees prior to organizational change have assumed that, if employees have positive attitudes and feeling about their jobs, they can accept organizational change (Farndale et al., 2011; van der Voet and Vermeeren, 2017). In responding to the increasing importance of the pre-change internal context in fostering constructive employee responses to organizational changes, Bouckenooghe et al. (2009) have argued that psychological climate for change, which encourages participation in decision making, is a precondition for inducing employee commitment, which in turn contributes to acceptance and support for organizational changes.

The central proposition of the social identity theory is that, through the perception of belongingness to a group, individuals define themselves in terms of their group membership and ascribe the characteristics that are typical of the group to themselves (Van Knippenberg, 2000). In other words, the more an individual identifies with the group, the more likely the individual is to behave complying with the group’s beliefs, norms, and values, and generally to behave in the ways expected by the group (Dutton et al., 1994). In general, this identification with the group (or organization) may induce individuals to take the group’s perspective and to experience the group’s goals and interests as their own. The relationship between identification with the organization and motivation to exert effort for the benefits of the organization may be positive especially when there are contextual factors making the social identity salient (Van Knippenberg, 2000). Encouraging members to speak up concerning work-related issues, for instance, may make public sector employees aware that they belong to the organization and have the duty to achieve the organization’s goals and work for the organization’s benefits (i.e., organizational change). Meyer and Parfyonova (2010) have further argued that normative commitment induces an individual’s desire to do the right thing beneficial for the organization, rather than compelling an individual to do things to avoid negative outcomes. Therefore, normative commitment, resulting from the perception of voice climate, involves a form of motivation to act in a manner that satisfies the norms and expectations of the organization. Even if the proposed change initiatives involve the organizational restructuring or removal of valued personal interests, it is plausible that individuals with higher normative commitment will react positively toward organizational change.

Based on the above discussion, we argue that psychological voice climate makes employees identify with the organization and produce normative commitment to do the right things that may benefit the organization—that is, increase acceptance and support for organizational changes. Several studies have also put emphasis on the role of the motivational base of public sector employees prior to organizational change (Farndale et al., 2011; van den Heuvel et al., 2017). These studies assume that, if employees have positive attitudes toward their organization, they will hold positive views toward organizational change. Therefore, we hypothesize that normative commitment will mediate the effect of psychological voice climate on individual change readiness.

Hypothesis 2: Normative commitment will mediate the relationship between psychological voice climate and individual change readiness.

### Moderated Mediation of Work Engagement

Work engagement, defined as the degree of vigor, dedication, and absorption that one experiences at work, represents the employee’s perceived contribution to the organization and the personal challenge that he or she derives from work (Macey and Schneider, 2008; Alarcon et al., 2010). Highly engaged individuals have more energy and persistence to complete their assigned tasks, such as successfully implementing the needed strategies (Demerouti and Cropanzano, 2010). In essence, work engagement can provide additional energy for employees in demanding situations (Schaufeli and Bakker, 2004). This energy may be viewed by employees as a resource when considering their reactions to organizational change initiatives. Employees with higher work engagement are likely motivated to utilize these resources when under stress (Bakker et al., 2007). One way to utilize work-related resources may be to engage in a change initiative. Thus, it is likely that highly engaged employees will enhance their change readiness.

However, work engagement is likely not enough to ensure success within a changing environment. Hallberg and Schaufeli (2006) have argued that, rather than just the opposite of job burnout, work engagement is a conceptualization of optimal functioning to help employees cope with organizational change. A growing number of studies indicate the possibility that work engagement serves a moderating role by attenuating or enhancing the relationships of job characteristics and work experiences with job outcomes (e.g., Shuck and Reio, 2014; Gordon et al.,2015). Employees with higher work engagement are thought to appraise their ability to meet their work demands positively, believe in good outcomes, and believe that they can satisfy their needs by fully engaging in their roles played in the organization (Knight et al., 2017). This may be particularly true for those engaged employees who feel obligated to do the right thing to repay for the existence of voice climate and exert more energy to support change initiatives in a changing context. Thus, this current study further explores whether work engagement moderates the indirect effects of psychological voice climate on individual change readiness through normative commitment.

Hypothesis 3: The indirect effects of psychological voice climate on individual change readiness will be stronger for employees with higher work engagement.

## Materials and Methods

### Organizational Context

The organizational change under investigation occurred in a government-owned institute of research and development in Taiwan. This organization is responsible for the development and design of technological instruments and integration systems. The organization had engaged in organizational restructuring in the pursuit of efficiency and the quality of services. Due to the nature of the organizational restructuring, employees would be directly affected in their almost daily operations. Of particular importance, the issues of job security and implications for status had become some concerns for these employees.

The change management project was sectioned into the design of new structures; the announcement of new working practices, procedures, and systems; and actual implementation time periods. Despite the fact that employees had no influence on the government’s decisions to implement the restructuring initiative, they had been encouraged to participate in a range of activities surrounding the work-related issues before the change initiative. After completing the design of new structures, top management officially informed employees that the restructuring would be implemented. Hence, this organization provides a unique opportunity for examining the influence of the pre-change internal context (i.e., psychological voice climate) on individual readiness for the planned organizational change.

### Sampling Procedure and Characteristics

Since the study did not involve animal experiments or human clinical trials, ethical approval was not required for this study but permission to proceed was obtained from the relevant change program managers who were assured that ethical principles would be followed. Before conducting the two-wave survey, employees were informed about the objectives of the study by their supervisors. They were assured that their participation was voluntary. At all times, confidentiality was maintained surrounding the employees involved and the information they disclosed. Being placed under no ‘undue pressure’ to participate, respondents held the right to not answer all questions. A covering letter was attached to each questionnaire emphasizing these points for all participants. Hence, we state that we conformed to the Helsinki Declaration concerning human rights and informed consent, and that we followed correct procedures concerning treatment of humans in research.

After receiving consent from the organizational change manager for the organizational restructuring initiative, we planned to collect data from employees at two points in time to reduce common method biases (Podsakoff et al., 2003) and improve methodological rigor in testing the causality of our research model (Wright et al., 2005).

The surveys were administered to the employees working in the organization. The data collection process was carried out in two waves. When the organizational change manager was planning for the redesign of organizational structure, we conducted the first wave of the survey. An email from the internal media was sent to 489 employees to complete the online survey concerning their perceptions of voice climate and demographic information. After 3 weeks, 405 employees had completed the survey, resulting in a response rate of 82.8%.

We conducted the second wave of the survey 6 months after the organizational change manager had announced the start of the organizational restructuring plan. The internal media was again used to connect with the participating employees from the first wave of the survey and collect information on normative commitment, individual change readiness, and work engagement. It is worth noting that the number of potentially participating employees was reduced to 210 (i.e., retention rate = 51.9% at Time 2) due to voluntary or involuntary quit from the institute after the restructuring plan had been implemented. After deleting any incomplete, mismatched, or missing cases, the final sample consisted of 120 male (64.2%) and 67 (35.8%) female employees, making the effective return rate 89.0%. Of the 187 employees, 21.4% were under the age of 35, 59.4% were between 36 and 55 years old, and 19.3 were 56 years old or older. The average organizational tenure was 17.9 years (*SD* = 12.3). The majority of the participating employees had graduated from college (50.3%) or higher (41.1%).

### Measurement

#### Psychological Voice Climate

The psychological voice climate was assessed with a six-item scale adapted from Van Dyne and LePine (1998), and the items had been worded such that the organization as a whole was the referent (Frazier and Fainshmidt, 2012; Frazier and Bowler, 2015). At the first-wave survey, employee were asked to respond on a 5-point scale anchored by 1 (*strongly disagree*) to 5 (*strongly agree*). The sample item is “It is worthwhile for employees to speak up with new ideas or changes in procedures.” The six-item measure of psychological voice climate used in this study yielded an acceptable internal consistency (Cronbach’s α = 0.94).

#### Normative Commitment

We applied Meyer et al.’s (1993) six-item scale to assess the level of employees’ normative commitment, such as “I would not leave my organization right now because I have a sense of obligation to the people in it.” At the second-wave survey, employees assessed the level of normative commitment via these items anchoring at 1 (*strongly disagree*) to 5 (*strongly agree*). The reliability coefficient (Cronbach’s α) of the normative commitment scale was 0.86.

#### Individual Change Readiness

We adopted four items from Holt et al.’s (2007) change readiness scale to measure the level of individual readiness for change. At the second-wave survey, we asked employees to evaluate their levels of readiness for the change program via a 5-point Likert scale. The Cronbach’s α value was 0.79.

#### Work Engagement

The measures of work engagement were taken from Schaufeli et al.’s (2006) 9-item short Utrecht Work Engagement Scale. It consists of items on vigor, absorption, and dedication (e.g., “At my work, I feel bursting with energy,” “I get carried away when I am working,” and “I am proud of the work that I do”). At the second-wave survey, we asked employees to report their level s of work engagement via a 5-point Likert scale. The reliability estimate (Cronbach’s α) was 0.94.

#### Control Variables

Gender and organizational tenure are potential predictors of normative commitment (Mathieu and Zajac, 1990; Ang et al., 2003). Gender has also been found to be associated with work engagement, with women more engaged than men (Rees et al., 2013). Prior research has shown that change readiness varies with the level of education (Holt et al., 2007). Thus, we used gender, level of education, and organizational tenure as control variables in our statistical analysis to reduce the possibility of spurious relationships that are based on unmeasured variables.

## Results

### Descriptive Analysis

**Table 1** shows the means, standard deviations, and correlations for all the variables in the study. Psychological voice climate was positively related to normative commitment (*r* = 0.33, *p* < 0.01), work engagement (*r* = 0.34, *p* < 0.01) and individual change readiness (*r* = 0.34, *p* < 0.01). The normative commitment was also positively related to work engagement (*r* = 0.60, *p* < 0.01), and change readiness (*r* = 0.46, *p* < 0.01).

**Table 1:** Means, standard deviations, and correlations of variables.

	Means (*M*)	Standard Deviation *(SD)*	1	2	3	4	5	6	7
(1) Gender^a^ (T1)	0.65	*0.48*							
(2) Level of education^b^ (T1)	2.34	*0.63*	0.34**						
(3) Organizational tenure (T1)	17.93	*12.25*	–0.30**	–0.50**					
(4) Psychological voice climate (T1)	3.29	*0.71*	–0.13	0.19	–0.14	(0.95)			
(5) Normative commitment (T2)	3.85	*0.63*	–0.11	–0.15*	0.02	0.33**	(0.86)		
(6) Work engagement (T2)	3.85	*0.59*	–0.04	–0.24**	0.12	0.34**	0.60**	(0.94)	
(7) Individual change readiness (T2)	3.59	*0.54*	0.03	–0.05	–0.05	0.34**	0.46**	0.53**	(0.79)

*N = 187, ^∗^*p* < 0.05, ^∗∗^*p* < 0.01, Coefficient composite reliabilities are reported in the diagonal. T1 = first-wave employee survey; T2 = second-wave employee survey. ^a^0 = female, 1 = male. ^b^1 = senior high school (senior vocational school)/2 = bachelor degree /3 = master degree or doctoral degree.*

### Measurement Model

In the beginning, we used Mplus 8.0 to perform confirmatory factor analysis to verify the discriminate validity of the four constructs in the study. The three facets of work engagement loaded onto a general engagement factor, and all indicators were allowed to load on their respective factors. All factors were allowed to correlate to one another in the confirmatory factor analysis. Results revealed that a four-factor model was well-fitted (χ2 = 322.57, *df* = 146, *p* < 0.01; CFI = 0.91, SRMR = 0.07. RMSEA = 0.07). All factor loadings were statistically significant, with standardized loadings ranging from 0.70 to 0.88. These results confirmed the discriminant validity of the constructs in our model (Kline, 2016).

We also conducted Harman’s single-factor test on common method variance (CMV) and found that the fit indices were not adequate for the one-factor model (χ2 = 1154.29, *df* = 152, *p* < 0.01; CFI = 0.51, SRMR = 0.19. RMSEA = 0.19). Additionally, the four-factor model was better than one-factor model (Δχ2 = 831.72, *df* = 6, *p* < 0.01), indicating that CMV was not a pervasive problem in this study.

### Hypothesis Testing

As shown in **Table 2**, we conducted a series of linear regression models to test the proposed hypotheses. First, we entered all control variables and psychological voice climate into Model 1. Results showed that psychological voice climate was significantly related to normative commitment (β = 0.485, *p* < 0.01), supporting Hypothesis 1. Additionally, psychological voice climate was significantly associated with individual change readiness (β = 0.255, *p* < 0.01, Model 3). Model 4 further showed that both of psychological voice climate (β = 0.150, *p* < 0.01) and normative commitment (β = 0.345, *p* < 0.01) were significantly related to individual change readiness after controlling gender, level of education, and organizational tenure.

**Table 2:** Results of overall model.

Dependent variables	Normative	Individual	Individual	Individual	Individual	Individual
	commitment	change	change	change	change	change
		readiness	readiness	readiness	readiness	readiness
	
	Model 1	Model 2	Model 3	Model 4	Model 5	Model 6
Constant	–1.496	3.656	3.788	3.641	3.563	3.494
Gender^a^ (T1)	–0.096 *(*-*1.02)*	0.081 *(1.10)*	–0.008 *(*-*0.90)*	0.048 *(0.60)*	0.019 *(0.26)*	0.103 *(0.14)*
Level of education^b^ (T1)	–0.0139 *(*-*1.75)*	–0.032 *(*-*0.47)*	–0.068 *(*-*0.95)*	–0.024 *(*-*0.37)*	0.027 *(0.42)*	0.026 *(0.41)*
Organizational tenure (T1)	–0.004 *(*-*1.07)*	–0.003 *(*-*0.79)*	–0.002 *(*-*0.58)*	–0.002 *(*-*0.48)*	–0.003 *(*-*0.92)*	–0.019 *(*-*0.63)*
Psychological voice climate (T1)	0.485** *(6.09)*	⋅	0.255** *(4.69)*	0.150** *(2.83)*	0.010 *(2.90)*	0.132** *(2.61)*
Normative commitment (T2)		0.402** *(7.04)*		0.345** *(5.79)*	0.142* *(2.12)*	0.135* *(2.06)*
Work engagement (T2)					0.348** *(4.88)*	0.355** *(5.03)*
Normative commitment × Work engagement						0.273** *(3.41)*
*F*	11.00**	13.06**	6.10**	12.44**	15.73**	15.94**
Adj *R*^2^	0.18	0.21	0.09	0.24	0.32	0.36
∆*R*^2^				0.14**	0.09**	0.04**

*All regression coefficients are unstandardized and the value in the parenthesis is *t*-value. Model 4, 5, and 6 were compared with model 3 separately. T1 = first-wave employee survey; T2 = second-wave employee survey. ^∗^*p* < 0.05, ^∗∗^*p* < 0.01. ^a^0 = female, 1 = male. ^b^1 = senior high school (senior vocational school)/2 = bachelor degree/3 = master degree or doctoral degree.*

Then, we used the bootstrapping method suggested by Preacher and Hayes (2004) to examine the indirect effect of psychological voice climate on individual change readiness through normative commitment. After 5000 times bootstrapping, the results showed that the indirect effect from psychological voice climate to individual change readiness via normative commitment was 0.154 (95% confidence interval = [0.05, 0.26]), supporting Hypothesis 2.

To test Hypothesis 3 that predicted the moderating effect of work engagement on the indirect effect from psychological voice climate to individual change readiness via normative commitment, we adopted the second stage moderation model proposed by Edwards and Lambert (2007). Then, we entered the interaction term of normative commitment and work engagement into Model 6. Results showed that the interaction term was statistically significant (β = 0.273, *p* < 0.01). Following the recommendation of prior research, we further tested the conditional indirect effect (Preacher and Hayes, 2004; Hayes, 2013). We controlled conditional effect of work engagement on the relationship between psychological voice climate and individual change readiness. Results from 5000 times bootstrapping showed that conditional effect was 0.04 (95% confidence interval = [0.01, 0.09]). Specifically, when work engagement was high, the indirect effect was significant (indirect effect = 0.09, 95% confidence interval = [0.04, 0.17]); however, when work engagement was low, the indirect effect was not significant (indirect effect = -0.01, 95% confidence interval = [-0.06, 0.04]). The findings provided support for Hypothesis 3.

**FIGURE 2:**
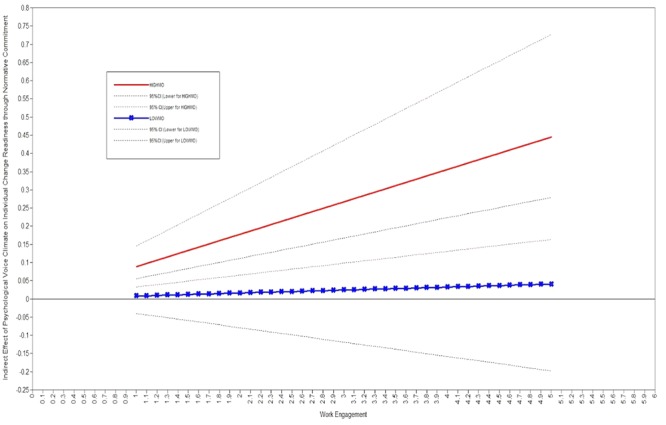
Indirect effects of psychological voice climate on individual change readiness through normative commitment conditional on work engagement.

In **Figure 2**, we plotted the conditional indirect effects of psychological voice climate on individual change readiness through normative commitment at various levels of work engagement (mean minus one standard deviation, mean plus one standard deviation). The solid lines were the estimates and the dashed lines represented for the upper and lower 95% confidence intervals. The 95% confidence interval of higher work engagement did not include 0; that is, the indirect effect was significant at the condition of higher work engagement. However, the 95% confidence interval of lower work engagement included 0; thus, the indirect effect was not significant at the condition of lower work engagement. Therefore, the indirect effects of psychological voice climate on individual change readiness was found stronger for employees with higher work engagement.

## Discussion

Why do public sector employees, surrounded by a positive voice climate, accept and support organizational changes? Grounded in the norm of reciprocity and the social identity theory, this research found that normative commitment mediates the relationship between psychological voice climate and individual change readiness; this indirect effect is conditional upon work engagement. By developing and examining these meditation and moderated-mediation mechanisms, thereby finding out answers to the above question, this research advances existing theories and practices in the field of public sectors’ change issues.

### Theoretical Implications

Since the development of the individual change readiness construct, extensive literature has outlined many factors reflecting beliefs, intentions, and attitudes to predicting an individual’s positive reactions toward the planned or unforeseen change initiatives (Choi and Ruona, 2011; Shah et al., 2017). Due to these advances, a strong consensus exists regarding the salient roles of the internal circumstances and the level of change readiness in understanding the processes that contribute to successful change implementation (Mento et al., 2002; Rafferty et al., 2013). Consequently, psychological climate for change was believed to influence employees’ attitudes toward organizational change (Bouckenooghe et al., 2009). Although psychological voice climate encourages employees to challenge the status quo to improve the situation, there is relative little known regarding how (i.e., underlying explanatory processes) and when (i.e., boundary conditions of the underlying processes) psychological voice climate is related to individual change readiness. Furthermore, the traditional perspective emphasizes the necessity of voice climate under which change occurs to foster voice behavior concerning change-related issues (Morrison et al., 2011), but ignores the essence of the conditions that are independent of organizational change and existed prior to the introduction of the change (Oreg and Sverdlik, 2011).

Responding to recent calls to explore whether and how pre-change internal contexts generate effects on employee responses to organizational change (van der Voet and Vermeeren, 2017), our research adopted the social identity theory and conducted a two-wave survey to analyze the relationship between pre-change psychological voice climate and individual change readiness occurring in the change process. The results confirmed the indirect effect of psychological voice climate on individual change readiness through normative commitment, implying that the pre-change internal context as perceived by the change recipients may be a key determinant for employees’ responses to organizational change. We suggest that pre-change voice climate could function as a change-conducive internal context and become an even more important success factor for realizing organizational change. Therefore, our research has increased our understanding of the relationship between psychological voice climate and individual change readiness.

Our research has also added knowledge to the public management literature about the mechanisms underlying the influential process of psychological voice climate on individual change readiness by incorporating the least studied component of organizational commitment (i.e., normative commitment). Although the significant effect of psychological voice climate on affective commitment has been examined in prior research (Farndale et al., 2011; McKay et al., 2013; Ditchburn and Hames, 2014), different mindsets of organizational commitment develop in different ways and have different implications for employee attitudes and behaviors (Powell and Meyer, 2004). In addition, substantial differences in employer-employee relationships and human resource management practices exemplify differences between public and private sector employment in Taiwan, which is likely to have different implications for the nature of commitment. Usually, the starting wage for employees in Taiwan’s public sector is higher than for those in the private sector; furthermore, given the security of employment and guaranteed pay increases, the public sector is a highly attractive career choice in Taiwan. As public organizations make large investments in employee compensation and benefits, we expect normative commitment to be a critical factor predicting public sector employees’ attitudes and behaviors. Therefore, this study drew on the social identity theory to understand the mediating role of normative commitment between psychological voice climate and individual change readiness.

Consistent with our expectations, the results confirmed the aforementioned hypotheses. Psychological voice climate was found to have a significant effect on normative commitment, which further mediated the effect of psychological voice climate on individual change readiness. In accordance with the social identity theory, when employees perceive that they have the opportunity to speak up their opinions and have their ideas taken into consideration, they feel and perceive themselves as belonging to this organization and produce greater normative commitment. The sense of moral duty to give back to the organization would foster employees to do the right thing on the behalf of the whole organization. During the organizational change process, the right thing means increasing change readiness. Based on the findings, we suggest that such a mechanism will be a powerful filter through which public sector employees will interpret their whole organizational environment while normative commitment will be a critical factor for transforming a pre-change voice climate into change readiness.

Finally, our research has essentially found that work engagement moderated the indirect effect of normative commitment between psychological voice climate and individual change readiness. Prior research mainly argued that work engagement may be important for countering potential dysfunctional attitudes and behaviors relevant to organizational change (van den Heuvel et al., 2010). The current study found that work engagement strengthened the effect of psychological voice climate on normative commitment as well as the subsequent individual change readiness. This finding is consistent with Schaufeli et al.’s (2002) argument that highly engaged employees have a sense of energetic and effective connection with their work activities, and they believe themselves able to deal with the demands of their job completely. In our study, when experiencing a high level of moral duty and a strong feeling of belonging to the organization as a result of perceptions of positive voice climate, highly engaged employees would utilize their energy to cope with the job demands accompanied by organizational change. Thus, our research provides new knowledge for the field of organizational change by clarifying the boundary condition in which psychological voice climate and normative commitment can effectively contribute to individual change readiness.

### Practical Implications

Our research has several practical applications for both public employees and public organizations undergoing organizational change. First, prior to the introduction of change initiatives, top management can induce higher levels of normative commitment by creating a sense of perceived influence on the decisions regarding the improvement of efficiency and effectiveness for the public organization. Combined with the symbolic meaning of demonstrating their confidence in the ability and wisdom of employees, managers need to convince employees that their opinions have been heard and taken into consideration. When employees feel confident that they can express their opinions successfully and doing so will not lead to their being punished or ignored, they become more willing to express their opinions and suggestions to do good for their organization.

Second, this study demonstrates the critical role of normative commitment in transforming psychological voice climate into readiness for change. Based on social exchange theory logic, as a result of the enhanced experience of inclusion, development, and personal growth from their organization, employees’ normative commitment increases (Yucel et al., 2014). As previously mentioned, more and more governments are facing increased financial deficits. Rather than offering economic and financial rewards, management of public sectors should develop a positive employee attitudinal approach using psychological motivation deriving from the climate that encourages participation in decision making, autonomy, and opportunities for personal growth and development (Edwards and Peccei, 2010).

Finally, our findings show that work engagement enhances the indirect effect of psychological voice positive on individual change readiness, which points to the need to support and cultivate engagement in the workforce. Following Christian et al.’s (2011) suggestion, managers can foster work engagement by designing jobs that include motivating characteristics and highlight the meanings of work in public organizations. In addition, because perceived organizational support is a significant predictor of work engagement (Saks, 2006), public organizations can implement organizational programs that address employees’ needs and concerns, express caring, and demonstrate support (e.g., flexible work arrangements), which may induce employees to reciprocate with higher levels of work engagement.

### Limitations and Directions for Further Research

Despite this study’s interesting findings and the contributions it makes to the field, it still has several limitations. First, the collection of data in one government-owned institute of research and development in Taiwan may potentially limit generalizability. Our observations should be interpreted with caution because public sectors *per se* and their employees in general may have different backgrounds and dimensions of cultures from public organizations in other countries. Thus, replicating the current study using data from other settings to see if the results hold in different kinds of public organizations and countries would be useful (Perry and Hondeghem, 2008). We also encourage researchers to explore the possibility of cultural differences occurring in psychological voice climate, normative commitment, work engagement, and individual change readiness.

Second, in the two-wave design using self-reported survey of employees, we had difficulty in contacting the leaving employees who had participated at the first-wave survey. The remaining employees were those who had “survived” from the organizational restructuring. We admit that, in such a sample, the responses potentially had a self-selection bias. Such a restriction of range would mean that the variance of the variables in this study has been underestimated, leading to a conservative estimation of their effect (Kalimo et al., 2003). The data revealed considerable variance in psychological voice climate (*SD* = 0.71), normative commitment (*SD* = 0.63), work engagement (*SD* = 0.59), and individual change readiness (*SD* = 0.54). These findings show that the respondents did not present themselves in a favorable or adverse way when assessing their own levels of these variables, indicating that the self-selection bias would not be serious in this study. Additionally, while the purposes of this study was to explore whether the pre-change internal context influences employees’ attitudes toward organizational change, the participants responding to both of the first-wave and second-wave surveys are likely to be the appropriate subjects for studying the experiences during the organizational change. However, we still suggest future studies use a multi-source data or objective indices related to experiences of significant organizational change to eliminate the self-selection bias to the minimum level.

Finally, an implication that arises from our review of the change readiness literature highlights the importance of considering what high and low levels of change readiness mean in an organizational setting (Rafferty et al., 2013). For example, Ford et al. (2008) have argued that low readiness for change may actually be an opportunity for an organization to identify the weaknesses in the execution of its organizational change plans. As such, there is need to examine the influence of individual change readiness on employee attitudes and job performance to fully understand the influence of psychological voice climate on change outcomes as a whole.

## Author Contributions

M-LW, C-HL, and M-SL: Contributed to study conception and design. M-LW and M-SL: Contributed to acquisition of data. C-HL and M-LW: Contributed to analysis and interpretation of data. M-LW, C-HL, and M-SL: Contributed to drafting of manuscript. M-LW and C-HL: Contributed in critical revision. M-LW and C-HL: Contributed to approval of the version of the manuscript to be published (the names of all authors must be listed).

## Conflict of Interest Statement

The authors declare that the research was conducted in the absence of any commercial or financial relationships that could be construed as a potential conflict of interest.
